# Phenotypic and Transcriptomic Analyses Reveal the Cell Membrane Damage of *Pseudomonas fragi* Induced by Cinnamic Acid

**DOI:** 10.3389/fmicb.2021.796754

**Published:** 2022-01-04

**Authors:** Yuxiang Zhang, Jianping Wei, Hong Guo, Chen Niu, Yahong Yuan, Tianli Yue

**Affiliations:** ^1^College of Food Science and Technology, Northwest University, Xi'an, China; ^2^College of Food Science and Engineering, Northwest A&F University, Yangling, China

**Keywords:** cinnamic acid, *Pseudomonas fragi*, membrane injury, antibacterial mechanism, RNA-seq

## Abstract

Cinnamic acid (CA) is a safe and effective antimicrobial agent. The objective of this study was to reveal the antibacterial mechanism of CA against a food-derived *Pseudomonas fragi* 38-8, from the aspects of bacterial growth kinetics, cell membrane homeostasis, cell microstructure, and transcription. The minimum inhibitory concentration (MIC) of CA against *P. fragi* 38-8 was 0.25 mg/ml. CA retarded bacterial growth and induced a series of cell membrane changes. After CA treatment, cell membrane homeostasis was destroyed, which was evidenced by cell membrane depolarization, intracellular pH reduction, and intracellular ATPase activity decrease. Field emission scanning electron microscope (FESEM), transmission electron microscope (TEM), and confocal laser scanning fluorescence microscope (CLSM) realized the visualization of cell microstructure changes, showing cell death and morphological changes, such as cell rupture, shrinkage, and hollowness. RNA sequencing analysis further confirmed the effects of CA to the cell membrane, because of the significant enrichment of differentially expressed genes (DEGs) related to membrane. The results of the phenotype tests and RNA-seq both focused on cell membrane damage, which showed that CA exerted antibacterial effect mainly by acting on cell membrane.

## Introduction

The genus *Pseudomonas* can usually cause food spoilage by shorting shelf life of fresh products, especially during cold storage, with a high economic burden for food industries (Quintieri et al., 2020). Currently, more than 240 valid published and correct named species belong to the category of *Pseudomonas* (Parte et al., 2020), some of which have been identified as key players of food spoilage, including *Pseudomonas fragi* (Kolbeck et al., 2021). *Pseudomonas fragi* is a facultative anaerobic and Gram-negative bacterium that is able to grow between 2 and 35°C (Ercolini et al., 2010). Their high spoilage potential is due to extracellular proteases and lipases (Zhang et al., 2019b), which act on foods, resulting in changes in the physicochemical and organoleptic properties. This kind of psychrotrophic bacterium has often been detected in some spoiled milk, meat, and seafood (Papadopoulou et al., 2020), particularly in food in the cold chain, where the *P. fragi* occupies a dominant position. It is urgent to take effective measures against *P. fragi* to reduce the economic loss within the cold chain.

Traditional thermal treatment has limitations under cold conditions. As natural products, plant-derived polyphenols have been the focus of extensive research and play an important role in food. Its antimicrobial activity has been largely investigated against a wide range of microorganisms, including Gram-negative and Gram-positive bacteria (Daglia, 2012; Papuc et al., 2017). Among these phenolic compounds, cinnamic acid (CA) is a common representative of phenylpropane-derived compounds, which are in the highest oxidation state, and some authors have found that more highly oxidized phenols are more inhibitory (Daglia, 2012). Therefore, the cinnamic skeleton is considered an interesting scaffold for developing novel biologically active substance, and CA derivatives are also known for their antimicrobial activity (Guzman, 2014; Ruwizhi and Aderibigbe, 2020). Cinnamic acid, especially *trans*-cinnamic acid, has exhibited potent inhibitory effect on many food-derived microbial organisms, such as *Stenotrophomonas maltophilia* (Zhang et al., 2019a), *Listeria monocytogenes* (Wen et al., 2003), *Escherichia coli* (Ordoñez et al., 2021), *Aeromonas sobria* (Yilmaz et al., 2018), and *Alicyclobacillus acidoterrestris* (Cai et al., 2019). Cinnamic acid could also effectively control gray mold of table grapes in the postharvest period by inhibiting the growth of the pathogen and inducing resistance of the host (Zhang et al., 2015). Moreover, CA might have the potential to control *Pseudomonas aeruginosa* infections due to its anti-quorum sensing and anti-biofilm effects (Rajkumari et al., 2018).

Regarding the antimicrobial action mechanism of CA, some studies have reported that it exerted activity mainly through destroying the plasma membrane, damaging nucleic acids and proteins, and inducing intracellular reactive oxygen species (Zhang et al., 2015; Cai et al., 2019). However, most studies on antibacterial effect of CA are only for the determination of phenotypic indicators, and little is known about the mechanisms of action at the molecular level. Transcriptomics is increasingly applied to the exploration of antibacterial mechanisms. Lin et al. (2017) and Chueca et al. (2017) have used microarray technology to reveal the mode of action of cinnamaldehyde, carvacrol, and citral on *E. coli*, respectively. Park et al. (2019) have studied the antibacterial mechanism of erythorbyl laurate against *Staphylococcus aureus* through RNA-seq analysis. Also, Zhang et al. (2020a) have introduced RNA sequencing to characterize the transcriptional response of *S. aureus* to heat stress.

Through our extensive screening of natural products, CA has shown good antibacterial activity. Based on previous research, this study further explores the antibacterial mechanism of CA against *P. fragi*, mainly focus on the changes of cell membrane. We measured cell membrane potential, intracellular pH, intracellular ATPase indicators, and used various electron microscopes to visualize the changes in cell membranes. Finally, RNA sequencing analysis was used to comprehensively reveal the transcriptional response of *P. fragi* to CA exposure.

## Materials and Methods

### Reagents and Bacterial Strain

Cinnamic acid (CAS 140-10-3) was purchased from the Shanghai Yuanye Bio-technology Co., Ltd. (Shanghai, China), with HPLC purity ≥98%. A stock solution of CA was prepared according to our previous research (Zhang et al., 2019a).

The fluorescent probe bis-(1,3-dibutylbarbituric acid) trimethine oxonol (DiBAC_4_(3); HPLC purity ≥95%; Sigma, United States) was used for the determination of cell membrane potential. The fluorescent probe 5(6)-carboxyfluorescein diacetate N-succinimidyl ester (CFSE; HPLC purity ≥90%; Sigma, United States) was used for the measurement of intracellular pH. The ATPase assay kit, purchased from Nanjing Jiancheng Bioengineering Institute (Nanjing, China), was chosen to assess intracellular ATPase activity. The LIVE/DEAD® BacLight™ Bacterial Viability Kit (Molecular Probes, Invitrogen), consisting of SYTO 9 and propidium iodide (PI) dyes, was used to evaluate the integrity of cell membranes.

*Pseudomonas fragi* 38-8 was preserved in our laboratory, which was isolated from commercial frozen fish balls (Zhang et al., 2019b). After propagation in nutrient broth (NB; 10 g peptone, 5 g NaCl, and 3 g beef extract in 1 L distilled water) medium at 25°C, the bacterial solution was centrifuged at 5,000 *g* for 10 min and the supernatant was discarded. Pellets of the strain 38-8 were washed thrice with PBS (pH 7.0) and then resuspended in the buffer solution to obtain a bacterial suspension with a concentration of 10^8^ colony forming units (CFU)/ml for subsequent experiments.

### MIC Determination and Growth Curves

The minimum inhibitory concentration (MIC) of CA was determined using the microdilution method as described by Zhang et al. (2019a). Antibacterial assays were carried out in a 96-well microtiter plate, and the initial bacterial concentration was controlled at 10^6^ cells per well. Five final concentration gradients of CA were set as 0, 0.125, 0.25, 0.5, and 1 mg/ml. The lowest concentration of CA that caused no visible growth of *P. fragi* 38-8 was determined as MIC.

After obtaining the MIC, a series of growth curves were drawn to observe the growth response of the strain 38-8 to *CA*. The CA and bacterial suspension prepared in section “Reagents and Bacterial Strain” were added to special measurement plates, ensuring that the final concentrations of CA reached 0 (CK), 1/2MIC, MIC, 2MIC, and 4MIC, and the bacterial concentration remained at 10^6^ cells in the final reaction system. The inoculated plates were cultured and monitored by an automatic growth curve analyzer (Bioscreen, Finland) at 25°C for 78 h, and the optical density (OD) at 600 nm was automatically recorded every 2 h.

### Membrane Potential Measurements

The cell membrane potential of *P. fragi* 38-8 was detected in accordance with a modified method of Sánchez et al. (2010). Bacterial suspensions were adjusted to an OD_600_ of 0.5. Next, 125 μl of cell suspensions was placed in a black 96-well microtiter plate for 30 min at 25°C. Subsequently, 1 μmol/L probe DiBAC_4_(3) was added, and the mixture was incubated at 25°C for 30 min. The CA at concentrations of 0 (CK), MIC, 2MIC, and 4MIC were prepared to treat the bacterial cells, and fluorescence intensity was measured after mixing for 5 min. All measurements were conducted by using a multi-function microplate detector (Spectra Max M2, Molecular Devices, United States), and the excitation/emission bandpass wavelengths were 492/515 nm. The background group without cells was set to correct the results.

### Changes in Intracellular pH

The measurement method of intracellular pH was based on previously published protocols (Breeuwer et al., 1996) with some modifications. Bacterial cells were first washed once with 50 mmol/L potassium phosphate buffer, and then washed twice with 50 mmol/L HEPES buffer (containing 5 mmol/L EDTA, pH 8.0). After centrifugation (5,000 *g*, 10 min), the bacterial pellet was suspended in 20 ml of HEPES buffer with 3 μmol/L CFSE probe and incubated at 25°C for 30 min. Cells loaded with probe were centrifuged, and washed with 50 mmol/L potassium phosphate buffer (containing 10 mmol/L MgCl_2_, pH 7.0) and then resuspended in 10 ml of this buffer. Afterward, 10 mmol/L glucose was added to eliminate the non-conjugated fluorescent probe (25°C, 30 min). Finally, the bacterial cells were washed twice with 50 mmol/L potassium phosphate buffer and resuspended in this solution.

Bacterial suspension prepared above was placed in a 96-well plate and treated with different concentrations of CA [0 (CK), MIC, 2MIC, and 4MIC] at 25°C for 30 min. The fluorescence was detected with a multi-function microplate detector (Spectra Max M2, Molecular Devices, United States). The measurement parameters were set to two excitation wavelengths of 490 and 440 nm, and one emission wavelength of 520 nm. Background fluorescence of cell-free wells was subtracted from each sample value. Meanwhile, a calibration curve was constructed with different pH buffers, according to a previous method (Shi et al., 2016a).

### Determination of Intracellular ATPase Activity

The effect of CA treatment on intracellular ATPase activity was examined by an ATPase assay kit. Bacterial suspension was adjusted to an OD_600_ of 0.5 and treated with a gradient of CA at 0 (CK), MIC, 2MIC, and 4MIC. After a full reaction (25°C, 30 min), the mixture was centrifuged at 5,000 *g* for 10 min. Pellets were washed thoroughly with physiological saline to eliminate the effects of residual bacteriostat. Then, cells were repeatedly frozen and thawed with liquid nitrogen at least five times to break the cells and dissolve the intracellular proteins. Next, the procedures were carried out following the operating instructions of the kit, which was mainly divided into two steps: enzymatic reaction and phosphorus determination process. When the operation was completed, the OD values of all samples were measured at 600 nm with a multi-function microplate detector. ATPase activity was calculated according to the formula in the instruction manual.

### Evaluation of Cell Membrane Integrity

#### Qualitative Observation

This study was conducted following the manufacturer instructions of the LIVE/DEAD® BacLight™ Bacterial Viability Kit. Briefly, bacterial cells were treated with CA at 0 (CK), MIC, 2MIC, and 4MIC for 30 min. A 2X working stain solution was obtained by mixing SYTO 9 and PI at a ratio of 1:1. The treated cells were stained, and then observed and photographed *via* a confocal laser scanning fluorescence microscope (CLSM; Andor Revolution WD, United Kingdom) to qualitatively characterize the damage of CA to cell membrane. The excitation/emission wavelengths were 480/500 nm for SYTO 9 and 490/635 nm for PI.

#### Quantitative Determination

The percentage of cell membrane damage was determined according to our previously published method (Zhang et al., 2019a). Live and dead bacteria were prepared and mixed in various proportions to make standard samples. Cells were treated with CA as described in section “Qualitative Observation.” All samples were stained with 2X working dye, and the fluorescence intensity was measured in the dark. Finally, the ratio of live cells was obtained by calculating the ratio of green fluorescence to red fluorescence.

### Assessment of Bacterial Ultrastructure

Morphological changes of bacteria were evaluated by field emission scanning electron microscopy (FESEM; Zhang et al., 2020b) and transmission electron microscopy (TEM; Zhang et al., 2019a). Cells were treated with 0 (CK) and MIC concentrations of CA at 25°C for 30 min. After centrifugation, the precipitates were washed with sterile PBS buffer and then fixed, rinsed, and dehydrated repeatedly following the reference method. The observations of surface morphology and internal structure were carried out using a Hitachi S-4800 FESEM (Hitachi Limited, Japan) and a JEM-1230 TEM (JEOL, Japan), respectively.

### RNA-Seq Analysis

In order to reveal the transcriptional changes of bacterial cells in response to CA, cultures of *P. fragi* 38-8 were treated with CA at MIC for 30 min (Marked as R), and untreated cells were used as controls (Marked as CK). All samples (R-1, R-2, R-3; CK-1, CK-2, and CK-3) were collected for subsequent steps of total RNA extraction, RNA purification, RNA quality testing, and library construction (Zhang et al., 2020a). Then, the library was quality-checked with Agilent 2,100 Bioanalyzer and ABI Step One Plus Real-Time PCR System, and RNA sequencing was performed on the Illumina HiSeq platform (Illumina, San Diego, CA, United States).

The raw data were filtered to remove those reads with low quality, contaminated adapters, and high content of unknown bases. *De novo* assembly of the resulting high-quality clean reads was conducted using Trinity (Haas et al., 2013). Assembled transcripts were then clustered and redundantly removed *via* Tgicl to obtain Unigene (Pertea et al., 2003). The Bowtie2 software was employed to align clean reads to reference gene sequence (Langmead and Salzberg, 2012), and RSEM was used to calculate the expression levels of genes and transcripts (Li and Dewey, 2011). The standardized method was FPKM (fragments per kilo-base of mRNA per million reads).

DEGs (differentially expressed genes) detection was performed with DEGSeq software package based on Poisson distribution. Value of *p* were corrected to Q-values according to Hochberg and Benjamini (1990) and Storey and Tibshirani (2003). To improve the accuracy of DEGs, those genes with a difference multiple of more than twice and Q-value ≤0.001 were screened as significantly DEGs. The Gene Ontology (GO) and Kyoto Encyclopedia of Genes and Genomes (KEGG) database were utilized to identify the main biological functions and metabolic pathways involved in the DEGs.

### Statistical Analysis

All tests were carried out at least in triplicate. The software SPSS 22.0 (SPSS Inc., Chicago, IL, United States) was used for statistical analysis, and Origin 9.0 (OriginLab, Northampton, MA, United States) was used for image processing. One-way ANOVA and Duncan’s multiple-range test were performed to evaluate the significance of the data (*p* < 0.05).

## Results and Discussion

### MIC and Growth Curves

CA exhibited a good bacteriostatic effect, and the MIC against *P. fragi* 38-8 was only 0.25 mg/ml.

**Figure 1:**
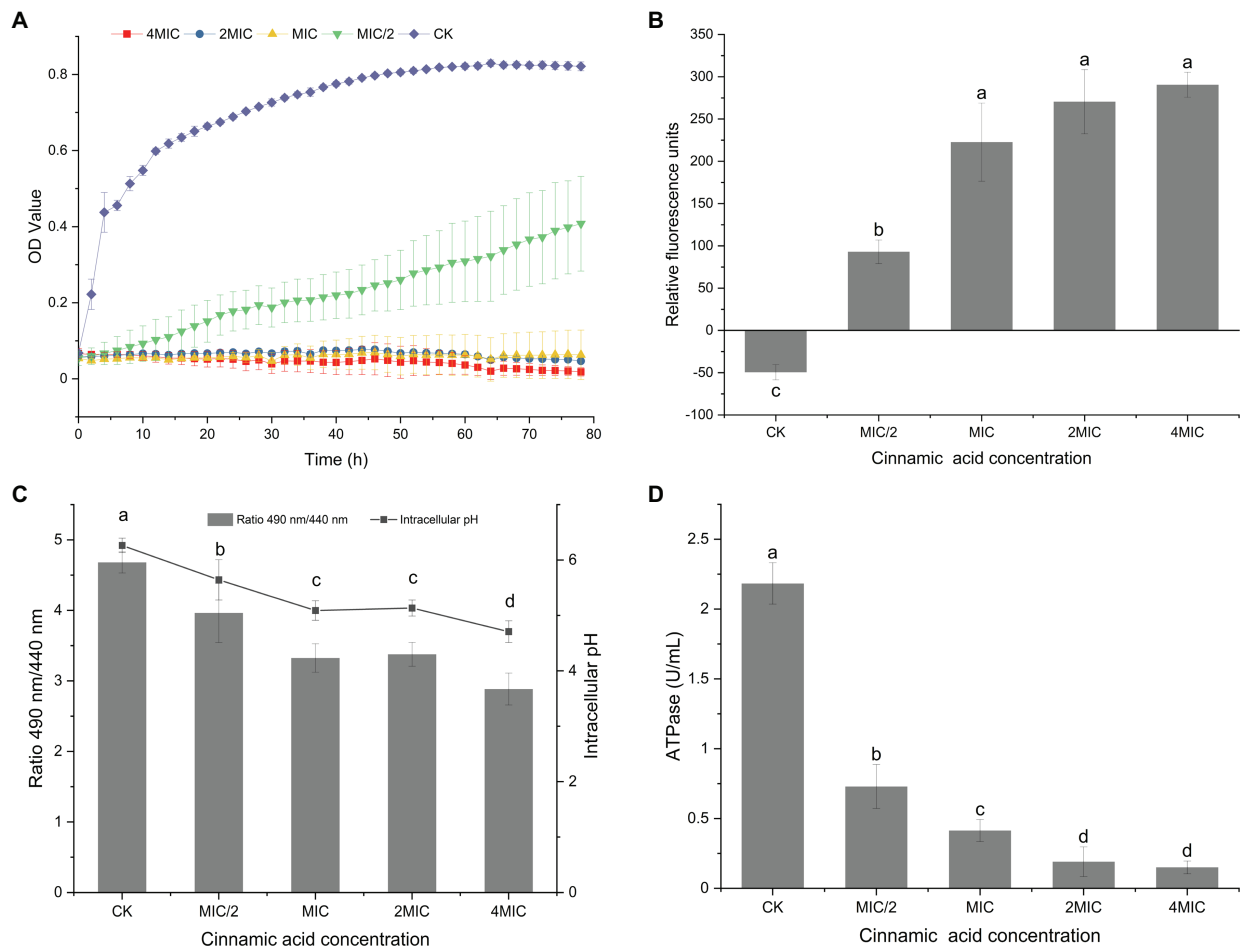
**(A)** Growth curves of *Pseudomonas fragi* 38-8 under different concentrations of cinnamic acid (CA); effect of CA on the membrane potentials **(B)**, intracellular pH **(C)**, and intracellular ATPase activity **(D)** of *P. fragi* 38-8. The letters over the bars indicate a significant difference (*p* < 0.05).

As shown in Figure 1A, growth of *P. fragi* 38-8 was inhibited by *CA*. When grown in medium without CA, the strain multiplied rapidly and grew well, and the OD value reached about 0.45 (approximately half of the maximum OD) within 4 h. When treated with CA at 1/2MIC (0.125 mg/ml), the growth rate of *P. fragi* 38-8 dropped sharply. The strain grew slowly and only reached an OD value of 0.40 after 78 h. Once the concentration was greater than the MIC, CA showed complete growth inhibition. A published study has shown that CA also has a good antibacterial effect on *Alicyclobacillus acidoterrestris* (Cai et al., 2015). As an active natural product with high-efficiency bacteriostatic activity, it was necessary to conduct an in-depth study on the antibacterial mechanism of CA against *P. fragi* 38-8.

### Cell Membrane Homeostasis

#### Membrane Potential

The fluorescent probe DiBAC_4_(3) used in this experiment is a commonly used negatively charged anionic slow-reacting dye. It has no fluorescence, but when it binds to the protein in the cytoplasm, fluorescence appears. As shown in Figure 1B, a relatively balanced resting potential difference was maintained on both sides of the cell membrane when unstimulated. After treatment with CA, the cells underwent significant depolarization, as evidenced by a significant increase in relative fluorescence intensity.

Cell membrane potential is an important indicator of the state of the cell membrane. Many scholars have reported the effect of antibacterial agents on cell membrane potential. For example, Shi et al. (2016b) pointed out that ferulic acid could cause hyperpolarization of the cell membrane of *Cronobacter sakazakii*. A researcher analyzed the mechanism of different plant extracts to inhibit *Vibrio cholerae* and found that the extracts of basil, white sagebrush, and sweet acacia could induce hyperpolarization of cells, while the depolarization of the cells occurred after treatment with the extract of nopal cactus (Sánchez et al., 2010).

#### Intracellular pH

The intracellular pH of the bacteria without CA treatment was 6.26 (Figure 1C). After treatment with CA at a concentration of MIC/2, the intracellular pH was reduced to 5.64. As the concentration of CA increased, the fluorescence ratio and intracellular pH gradually decreased. When the CA concentration increased to 4MIC, the intracellular pH decreased to 4.71.

Intracellular pH is an indicator of cell homeostasis, which is essential for the control of many cellular processes, such as DNA transcription, protein synthesis, and enzyme activity (Breeuwer et al., 1996). Cells with intact cell membranes can maintain the internal pH through ion channels and pumps when the external pH value changes moderately (Fitzgerald et al., 2004). CA treatment broke the equilibrium state of cells, resulting in a reduction in intracellular pH. Similarly, many antibacterial agents can induce a decrease in intracellular pH, which often means damage to the bacterial cell membranes (Sánchez et al., 2010; Shi et al., 2016a).

#### Intracellular ATPase Activity

The ATPase activity in normal bacterial cells were the highest (Figure 1D). CA treatment reduced the ATPase activity, and with the increase of CA concentration, the ATPase activity decreased significantly. ATPase is a kind of enzymes that exists on tissue membrane and organelle membrane. It participates in the processes of material transport, energy conversion, and information transfer of cells, and plays an important role in maintaining normal physiological and metabolic activities of cells. CA treatment did cause the loss of intracellular ATPase and decrease its activity, thus destroying the homeostasis of cell membrane and interfering with intracellular energy metabolism.

### Cell Membrane Integrity

#### Fluorescence-Based Live/Dead Assay

The SYTO 9 green fluorescent dye that marks all cells is usually used to assess the total number of bacterial cells, while the presence of the red fluorescent dye PI, which only penetrates bacterial cells with damaged membranes, will weaken the green fluorescence of SYTO 9 (Berney et al., 2007).

**Figure 2:**
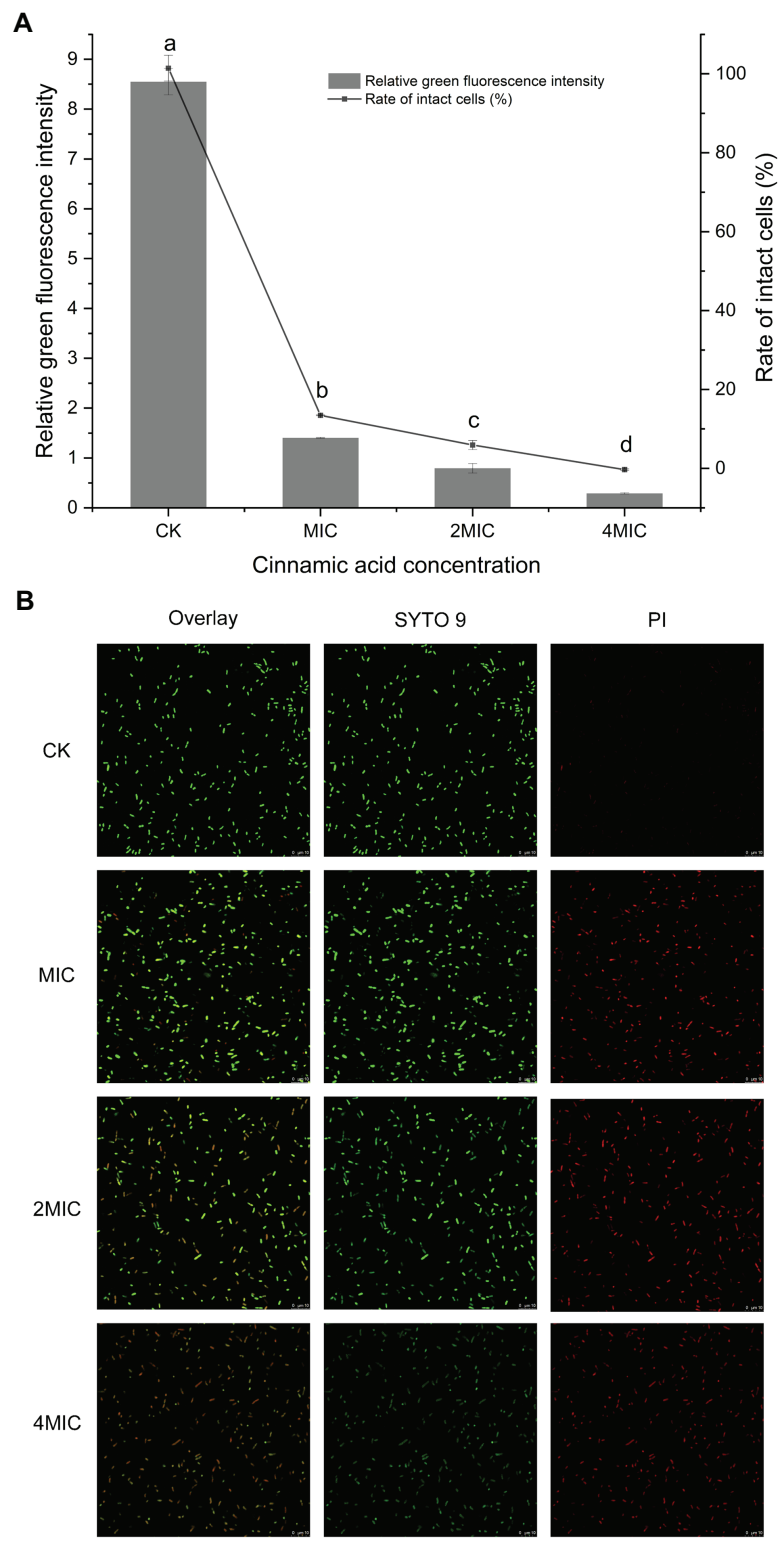
**(A)** Effect of CA on the membrane integrity of *P. fragi* 38-8. The letters over the bars indicate a significant difference (*p* < 0.05); **(B)** images of *P. fragi* 38-8 by CLSM.

Almost all cells in the control group showed green fluorescence (Figure 2B), indicating that the cells grew well and had intact cell membranes. The quantitative test also confirmed this result, as evidenced by the 100% intact cell (Figure 2A). When treated with different concentrations of CA, the membrane-damaged cells increased significantly. Furthermore, significant differences in the ratio of damaged cells appeared among the MIC, 2MIC, and 4MIC treatments, which caused 87, 94, and 100% of cell deaths, respectively (Figure 2A). Consistently, the strong red fluorescence positively correlated with CA concentrations was observed in CLSM images, while the green fluorescence gradually weakened and disappeared as the exposure concentration increased (Figure 2B). The introduction of CA induced the damage of cell membranes, leading to the death of bacteria, and the bacterial mortality was proportional to the CA concentration.

#### Ultrastructural Changes

**Figure 3:**
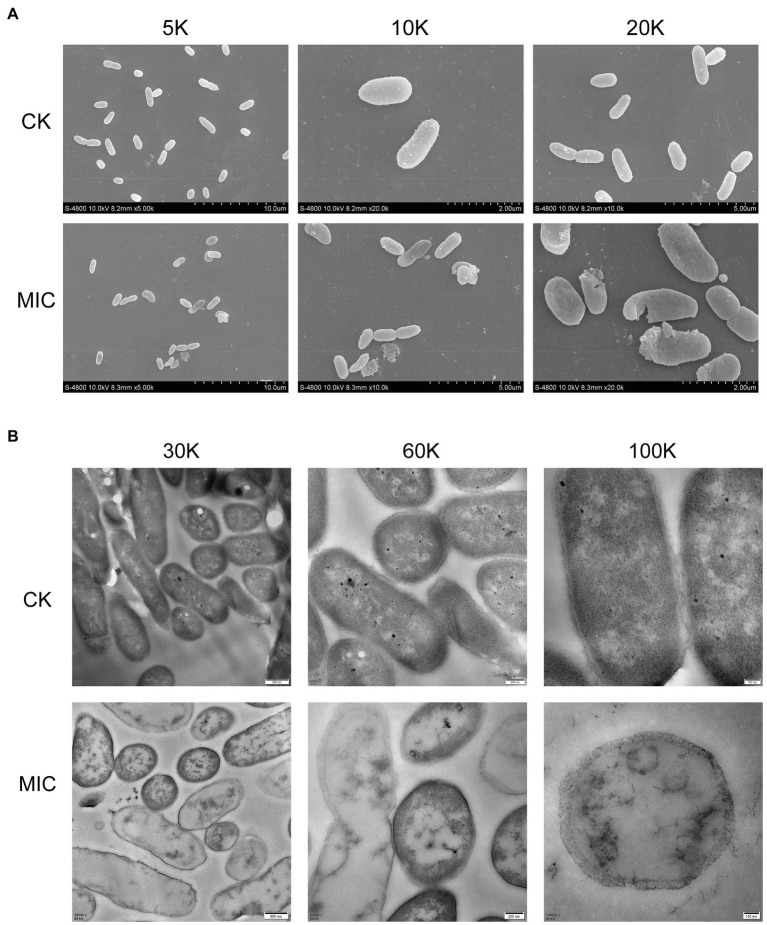
Morphological changes **(A)** and internal structural changes **(B)** of *P. fragi* 38-8 before and after CA treatment.

FESEM images (Figure 3A) showed that cell membrane changes occurred after treatment with *CA*. Untreated *P. fragi* cells presented typical rod-shaped structures with full shape and shiny surface. When exposed to CA, the cells became shriveled, collapsed, severely deformed, and even fragmented. These results prove that CA changes the surface morphology and causes irreversible damage to cell walls and membranes. According to the literatures, similar morphological changes of bacterial cells have also appeared in the treatment with other plant-derived antibacterial agents (Chen et al., 2017; Cai et al., 2019).

TEM images (Figure 3B) demonstrated that CA could affect the internal ultrastructure of cells. Untreated cells had normal morphology, clear boundaries, and uniform and dense cytoplasm. CA blurred the cell boundaries and made the cell contents flow out; the cytoplasm was destroyed and lost its density, and cells showed varying degrees of hollowness.

Deformation of the physical structure of the cell causes swelling and instability of the membrane, even a relatively slight change to the structural integrity can adversely affect cell metabolism and lead to cell death (Chen et al., 2017). Such changes usually affect the fluidity and permeability of the cell membranes, which are often reflected in the loss and leakage of various important components in cells, such as ions, ATP, nucleic acids, and amino acids. Combined with the results in section “Cell Membrane Homeostasis,” CA treatment not only caused changes in cell morphology and structure, but also affected cell membrane potential, changed intracellular pH, and caused intracellular ATPase leakage. All these factors that ultimately led to the inactivation of the bacteria were attributed to the destruction of the cell membrane. Therefore, the major target site of CA to exert antibacterial activity was probably the cytoplasmic membrane.

### Transcriptomics Analysis of Antibacterial Molecular Mechanism

In order to clarify the mechanism of CA on *P. fragi* at the genetic level, the transcripts of *P. fragi* 38-8 before and after CA treatment were sequenced, focusing on the damage to the cell membrane.

#### Overall Analysis

A total of 8.19 Gb of data were measured for all six samples by transcriptome sequencing (Table 1). After quality filtering raw reads, over 9.01 M clean reads with Q30 value for over 96.3% of the sequence were obtained for all samples. Moreover, at least 86.1% of clean reads successfully aligned to the reference gene sequence.

**Table 1:** Summary of RNA-seq data of all samples.

Parameter	Sample					
	CK-1	CK-2	CK-3	R-1	R-2	R-3
Total raw reads (M)	9.8	9.8	9.8	9.8	9.8	9.8
Total clean reads (M)	9.21	9.16	9.16	9.06	9.05	9.01
Total clean bases (Gb)	1.38	1.37	1.37	1.36	1.36	1.35
Clean reads Q20 (%)	98.91	98.84	98.92	98.77	98.81	98.77
Clean reads Q30 (%)	96.51	96.31	96.59	96.3	96.38	96.3
Clean reads ratio (%)	93.99	93.56	93.51	92.47	92.42	91.97
Total mapping (%)	88.28	86.1	87.78	86.5	86.76	88.55
GC (%)	58.39	58.74	58.26	57.98	58.07	58.06

Total raw reads refer to the number of reads before filtering; total clean reads refer to the number of reads obtained after filtering; total clean bases refer to the number of bases after filtering; Q20 refers to the ratio of the number of bases with a quality value greater than 20 to the total number of bases in the filtered reads; Q30 refers to the ratio of the number of bases with a quality value greater than 30 to the total number of bases in the filtered reads; total mapping refers to the ratio of Clean Reads aligned to the gene sequence; and GC refers to the percentage of GC bases.

**Figure 4:**
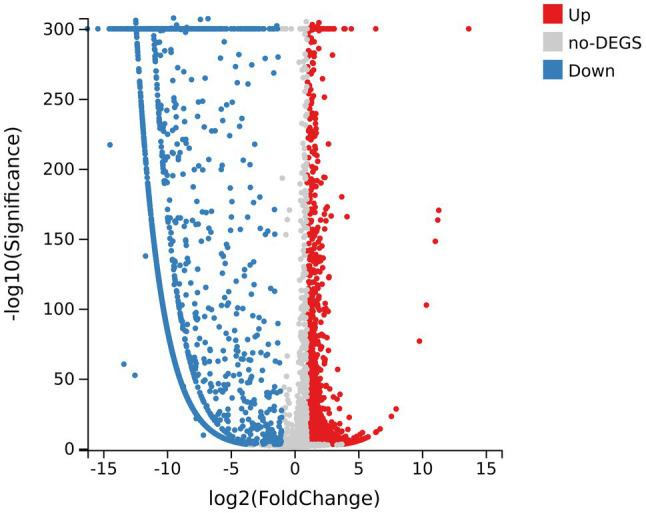
Volcano map of differentially expressed genes (DEGs) of samples. The abscissa axis represents fold change after log2 conversion; the vertical axis represents the significance value after the -log10 conversion. Red dots indicate the upregulated DEGs; blue dots indicate the downregulated DEGs; and gray dots indicate genes with no significant differences.

There were 5,024 expressed genes in all samples (Supplementary Figure 1), of which 3,793 DEGs appeared when comparing R group and CK group, including 1,335 upregulated DEGs and 2,458 downregulated DEGs (Figure 4). By the way, obvious differences existed between R group and CK group in terms of gene expression; while the parallelism of three replicates in each group was good, indicating that the sequencing results were reliable and available for subsequent analysis (Supplementary Figure 2).

#### GO Enrichment Analysis

**Figure 5:**
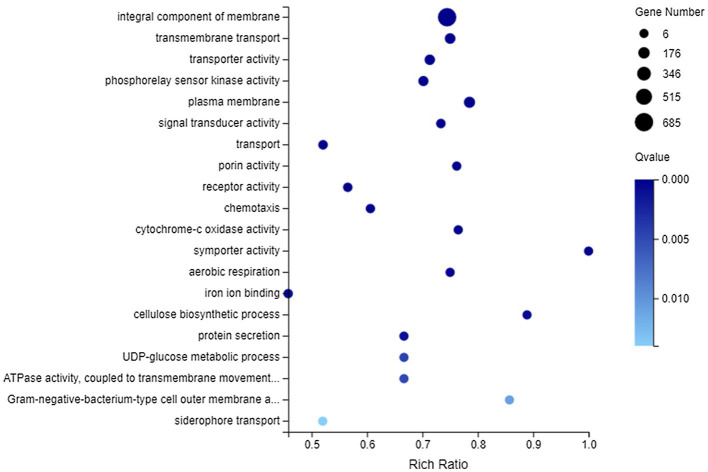
Gene Ontology (GO) enrichment of membrane-related DEGs. The vertical axis represents GO term and the abscissa axis represents the rich ratio.

Among all DEGs, there were 2,192 DEGs that could be annotated into 1,066 GO terms, which were mainly associated with catalytic activity, binding, cellular process, metabolic process, membrane, and membrane part, whether they were upregulated or downregulated (Supplementary Figure 3). Furthermore, GO enrichment analysis was performed on 820 DEGs (including 607 downregulated genes and 213 upregulated genes) matched to membrane. These DEGs could be enriched to 345 GO terms, of which 35 GO terms showed significant enrichment (Q value <0.05). Figure 5 lists the top 20 GO terms enriched by DEGs. It could be concluded that DEGs related to membrane were most significantly enriched (Q value <0.001) in integral component of membrane, transmembrane transport, transporter activity, phosphorelay sensor kinase activity, plasma membrane, signal transducer activity, transport, porin activity, receptor activity, chemotaxis, cytochrome-c oxidase activity, symporter activity, aerobic respiration, iron ion binding, and cellulose biosynthetic process.

#### KEGG Enrichment Analysis

After exposure to CA, a total of 1737 DEGs were enriched to 146 KEGG pathways, while the number of DEGs involved in each pathway was different, ranging from 1 to 310. The top 10 pathways in DEGs were Two-component system (310 DEGs), Microbial metabolism in diverse environments (267 DEGs), Biosynthesis of antibiotics (259 DEGs), ABC transporters (138 DEGs), Biosynthesis of amino acids (125 DEGs), Carbon metabolism (124 DEGs), Biofilm formation – *Pseudomonas aeruginosa* (72 DEGs), Quorum sensing (69 DEGs), Bacterial chemotaxis (67 DEGs), and Glyoxylate and dicarboxylate metabolism (66 DEGs). Besides, the KEGG enrichment analysis of 553 upregulated genes and 1,184 downregulated genes showed that 128 pathways and 144 pathways were, respectively, related to these genes, but none of them were significantly enriched (Supplementary Figure 4).

**Figure 6:**
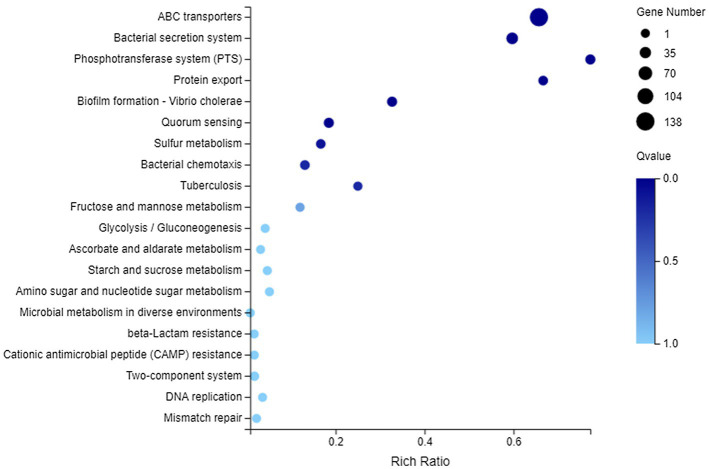
Kyoto Encyclopedia of Genes and Genomes (KEGG) enrichment of membrane transport-related DEGs. The vertical axis represents the name of pathway and the abscissa axis represents the rich ratio.

In terms of membrane transport, 194 DEGs were annotated to this category, covering 119 genes with downregulated expression and 75 genes with upregulated expression. They could be enriched to 23 KEGG pathways, and the first 20 pathways are shown in Figure 6. There into, significantly enriched pathways (Q value <0.05) included ABC transporters, Bacterial secretion system, Phosphotransferase system (PTS), Protein export, Biofilm formation-*Vibrio cholerae*, and Quorum sensing.

Cell membrane acts as a basic barrier between the cytoplasm and the external environment, maintaining a suitable internal condition for physiological metabolism and energy transduction (Sánchez et al., 2010). Once the cell membrane is destroyed, it will cause a series of dysfunctions in bacterial cell and make the cell inactive. Many previous studies have reported the destruction of cell membranes by antibacterial agents. Lin et al. (2017) have studied the transcriptome of *E. coli* regulated by cinnamaldehyde and found that flagellin and cell membrane may be the initial antibacterial target. In addition, cinnamaldehyde could also affect the synthesis and aggregation of cytoplasmic proteins, interfere with the metabolism of ATP, and lead to bacterial metabolic disorders and autolysis. When analyzing the mechanism of carvacrol and citral on *E. coli*, it was also concluded that they could cause membrane damage (Chueca et al., 2017). Guo et al. (2019) have confirmed the changes in cell membrane and cytoplasmic composition of Huyou essential oil-induced *Listeria monocytogenes* through morphological changes and RNA sequencing. At the same time, it has also pointed out that cell metabolism may be changed after treatment, and DEGs were enriched in protein export pathway, bacterial secretion systems, ABC transporter pathway, and quorum sensing. These findings were very similar to our results. Park et al. (2019) have conducted a transcriptomic analysis of *S. aureus* under the stress condition of antibacterial erythorbyl laurate. The results have indicated that the expression of genes related to cell growth was downregulated, mainly in energy metabolism, nucleic acid metabolism, translation, cell division, and transport, while the expression of genes related to cell wall stress stimulation was upregulated. However, in this study, fewer genes were upregulated after CA treatment, which may be related to the CA concentration. Combined with section “Cell Membrane Integrity,” the proportion of intact cells under MIC concentration treatment was only 13%. At this time, the amount of downregulated genes caused by cell damage was far more than the amount of upregulated genes induced by stress response.

## Conclusion

In summary, CA is a promising compound for inactivating *P. fragi*, with an MIC of 0.25 mg/ml. It exerted the antibacterial effect by interfering with the cell membrane homeostasis and destroying cell membrane integrity as evidenced by cell membrane depolarization, reduction of intracellular pH, loss of intracellular ATPase activity, and morphological changes of cell membrane. Exploiting this fact, RNA-seq analysis mainly focused on the DEGs related to cell membrane and found that these DEGs showed significant enrichment, indicating that the cell membrane was damaged, which was also consistent with the phenotypic changes. Therefore, we believed that the destruction of cell membrane by CA was the most direct and core cause of bacterial cell death. Moreover, some DEGs were closely related to energy metabolism, bacterial motility, biofilm formation, and quorum sensing, which indicated that the binding site of CA in *P. fragi* may not be limited to cell membrane. It is likely that a series of physiological metabolic disorders caused by cell membrane damage together lead to cell inactivation. Additional experiments, such as those assessing quorum sensing inhibition, proteomic changes, and metabolic responses, should be carried out in the future to fully reveal the mode of action of CA on *P. fragi* from multiple angles.

## Data Availability Statement

The datasets presented in this study can be found in online repositories. The names of the repository/repositories and accession number(s) can be found at: NCBI – OK559600, PRJNA772740 SRA – SAMN22408735, SAMN22408736, SAMN22408737, SAMN22408741, SAMN22408742, and SAMN22408743.

## Author Contributions

YZ, JW, and TY conceived and designed the experiments. YZ and JW performed the experiments and analyzed the data. YZ, JW, and HG searched the literature and wrote sections of the manuscript. CN and YY organized the framework and revised the manuscript. All authors contributed to manuscript revision, read, and approved the submitted version.

## Funding

The work was supported by the National Natural Science Foundation of China (32001673) and the Young Talent fund of University Association for Science and Technology in Shaanxi, China (20210207).

## Conflict of Interest

The authors declare that the research was conducted in the absence of any commercial or financial relationships that could be construed as a potential conflict of interest.

## Publisher’s Note

All claims expressed in this article are solely those of the authors and do not necessarily represent those of their affiliated organizations, or those of the publisher, the editors and the reviewers. Any product that may be evaluated in this article, or claim that may be made by its manufacturer, is not guaranteed or endorsed by the publisher.
