# Supplementary material for: Phenotypic and Transcriptomic Analyses Reveal the Cell Membrane Damage of Pseudomonas fragi Induced by Cinnamic Acid
Source: Front Microbiol. 2022 Jan 4;12:796754. doi: 10.3389/fmicb.2021.796754 (PMC8764163; doi:10.3389/fmicb.2021.796754)
Supplement: Supplementary file 1 [file Table_1.DOCX]

Supplementary Material

#
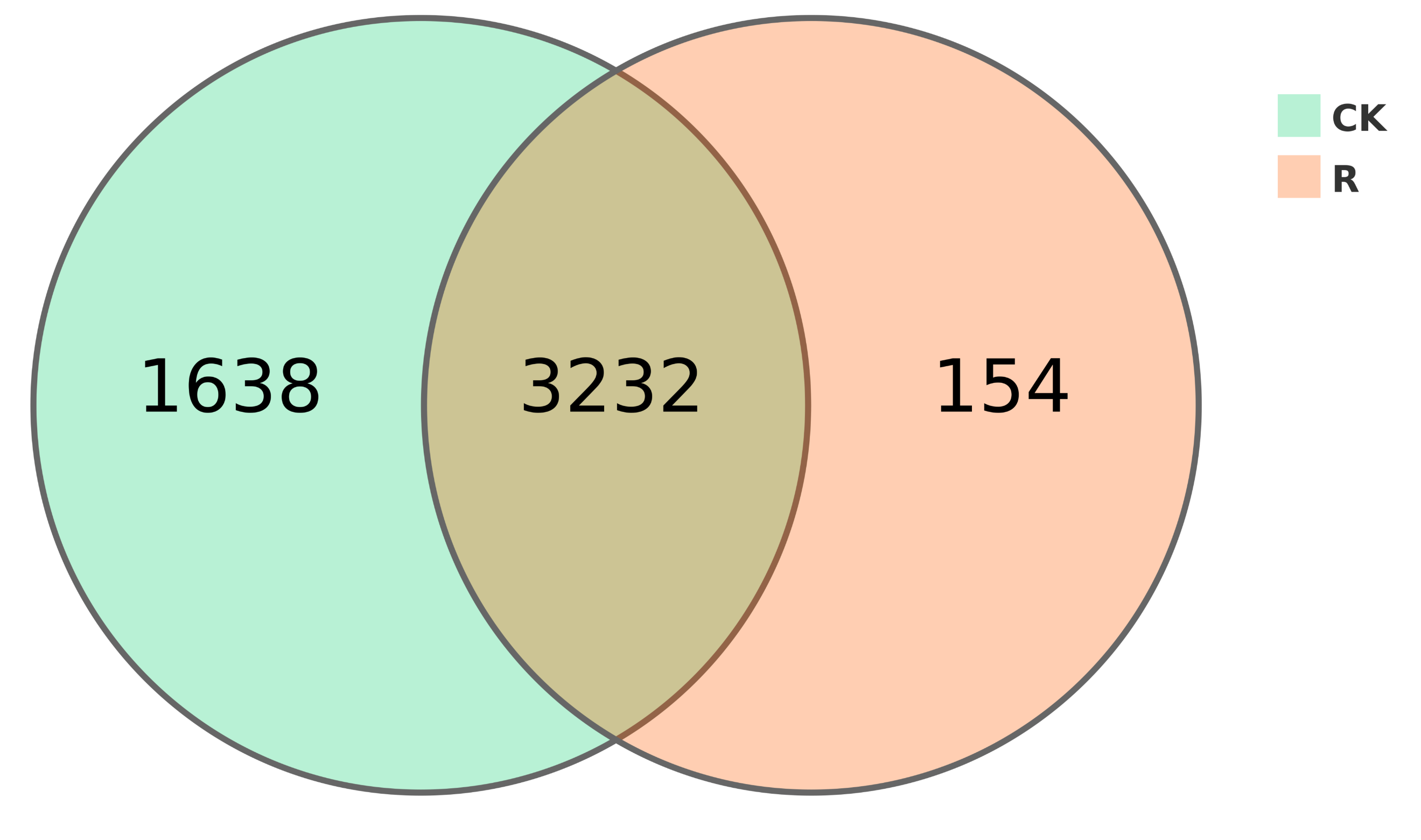
Supplementary Figures

**Supplementary Figure 1.** Veen diagram of gene expression. CK represents the samples without cinnamic acid; R represents the cinnamic acid treated sample.

**
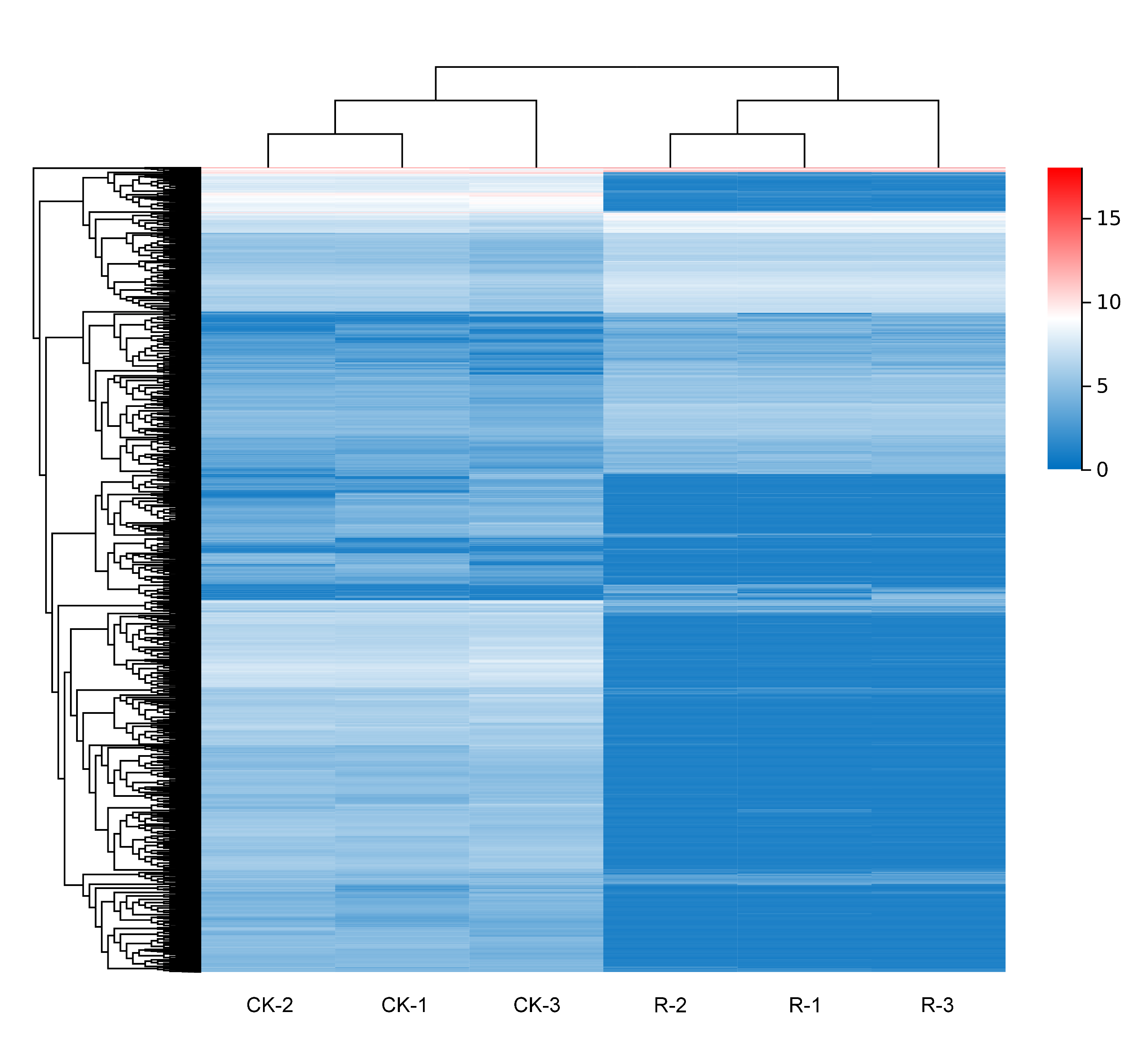
**

**Supplementary Figure 2.** Cluster heat map of differential gene expressions. CK represents the samples without cinnamic acid; R represents the cinnamic acid treated sample. Redder indicates a higher expression level; bluer indicates a lower expression level.


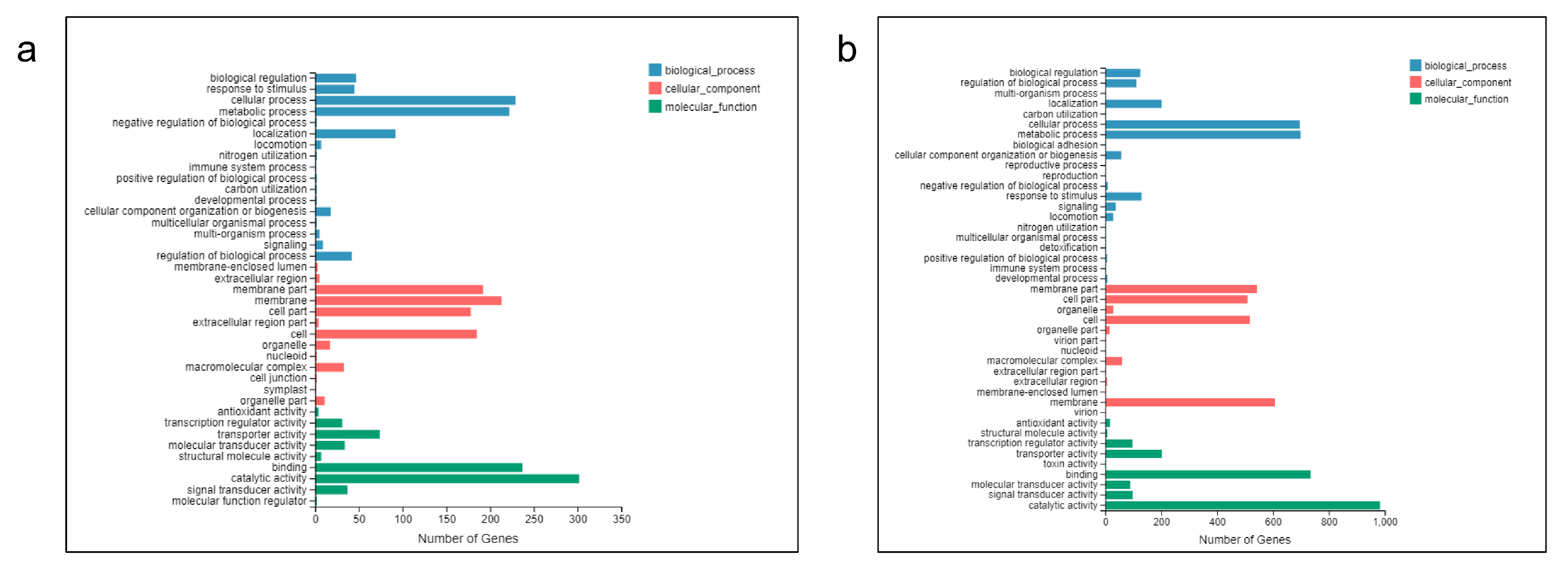


**Supplementary Figure 3.** GO functional classification of up-regulated (a) and down-regulated (b) differentially expressed genes (DEGs). The vertical axis represents GO term; the abscissa axis represents the number of DEGs of each GO term.

**
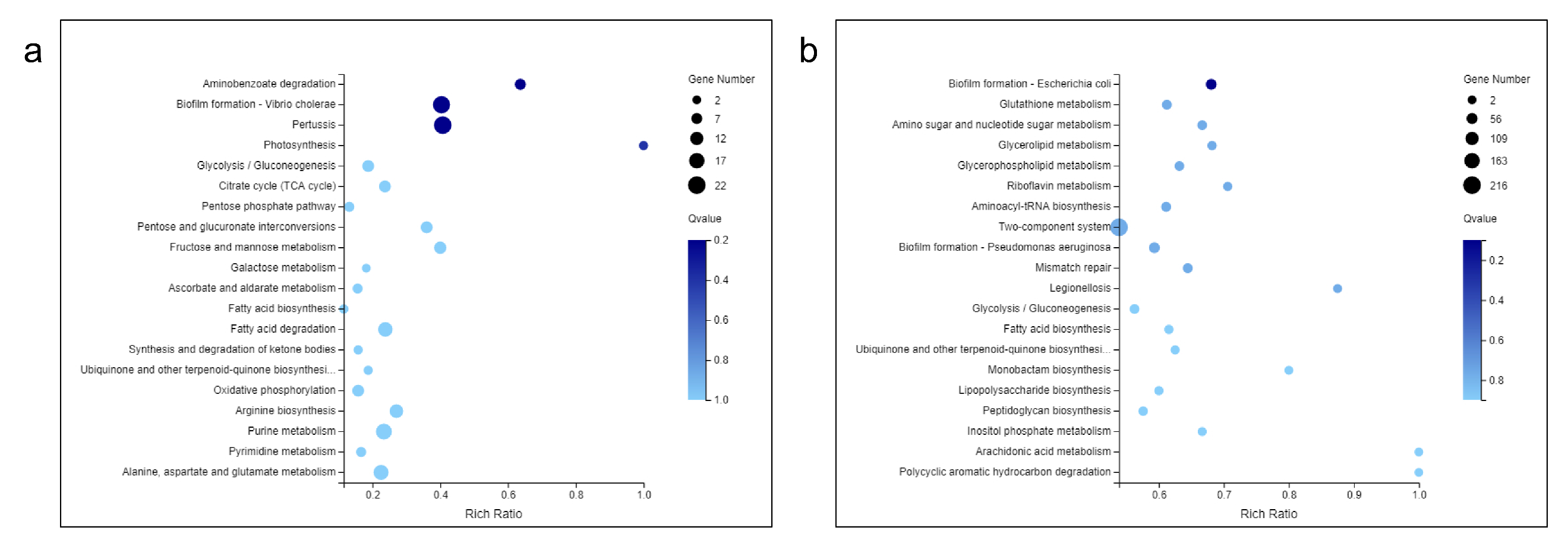
**

**Supplementary Figure 4.** KEGG enrichment of up-regulated (a) and down-regulated (b) differentially expressed genes (DEGs). The vertical axis represents the name of pathway; the abscissa axis represents the rich ratio.
